# Enhanced insights from human and animal host-associated molecular marker genes in a freshwater lake receiving wet weather overflows

**DOI:** 10.1038/s41598-019-48682-4

**Published:** 2019-08-29

**Authors:** Warish Ahmed, Sudhi Payyappat, Michele Cassidy, Colin Besley

**Affiliations:** 1 CSIRO Land and Water, Ecosciences Precinct, 41 Boggo Road, Dutton Park, QLD 4102 Australia; 2 Sydney Water, 1 Smith Street, Parramatta, NSW 2150 Australia

**Keywords:** Microbiology, Water microbiology, Environmental sciences

## Abstract

This study investigated the magnitude of wet weather overflow (WWO)-driven sewage pollution in an urban lake (Lake Parramatta) located in Sydney, New South Wales, Australia. Water samples were collected during a dry weather period and after two storm events, and tested for a range of novel and established sewage- [*Bacteroides* HF183, crAssphage CPQ_056 and pepper mild mottle virus (PMMoV)] and animal feces-associated (*Bacteroides* BacCan-UCD, cowM2 and *Helicobacter* spp. associated GFD) microbial source tracking marker genes along with the enumeration of culturable fecal indicator bacteria (FIB), namely *Escherichia coli* (*E. coli)* and *Enterococcus* spp. The magnitude of general and source-specific fecal pollution was low in water samples collected during dry weather compared to storm events. The levels of HF183, crAssphage and PMMoV in water samples collected during storm events were as high as 6.39, 6.33 and 5.27 log_10_ GC/L of water, respectively. Moderate to strong positive correlations were observed among the quantitative occurrence of sewage-associated marker genes. The concentrations of HF183 and PMMoV in most storm water samples exceeded the risk benchmark threshold values established in the literature for primary contact recreators. None of the samples tested was positive for the cowM2 (cow) marker gene, while BacCan-UCD (dog) and GFD (avian) animal-associated markers were sporadically detected in water samples collected from both dry weather and storm events. Based on the results, the ongoing advice that swimming should be avoided for several days after storm events appears appropriate. Further research to determine the decay rates of sewage-associated marker genes in relation to each other and enteric viruses would help refine current advice. Microbial source tracking approaches employed in this study provided insights into sources of contamination over currently used FIB.

## Introduction

The primary risk associated with fecal pollution is human health. Fecally contaminated water may contain numerous pathogens with low infectious doses with the potential to cause diseases in recreational water users^1^. A recent study estimated the amount of recoverable feces using country-specific human and animal population^2^. In 2014, the total mass of feces was 3.9 × 10^12^ kg/year and is anticipated to reach at least 4.6 × 10^12^ kg in 2030^2^. This increase will result in increased fecal contamination of catchment waters. Between animal and human populations, human feces pose a greater risk due to the presence of enteric viruses such as norovirus^3^ and enteroviruses^4^, and protozoa such as *Cryptosporidium parvum* and *Giardia lamblia*, in the feces of infected individuals^5,6^. Cattle, swine and poultry also harbor a number of pathogens, such as *Campylobacter jejuni*, *C. parvum*, *G. lamblia*, *E. coli* (EC) O157:H7 and other clinically significant EC^7–9^.

To understand the human health risks associated with fecally contaminated water, it is ideal to screen for pathogens. However, it is impractical, time-consuming, cost-prohibitive and technologically challenging to screen water samples for all potential pathogens. As an alternative, fecal indicator bacteria (FIB), such as EC, *Enterococcus* spp., (ENT) *Clostridium perfringens*, and bacteriophage, have been used to predict the potential presence of pathogens in water sources. The application of FIB in monitoring water quality has been criticized because FIB are able to survive and grow in various environments, such as soils, sands, sediments, and aquatic vegetation under favorable conditions^10–13^. Studies have also reported poor correlations between FIB and pathogens in sewage, recreational water, drinking water reservoirs, stormwater, and groundwater^14–17^. Finally, FIB monitoring does not provide information on the source of contamination. Without knowing the contamination sources, incorporating best management practices (BMPs) and remediation efforts are difficult.

In response to the shortcomings of FIB, researchers have developed microbial source tracking (MST) management tools that can be used to identify the sources of contamination. Currently, MST tools can be used to identify fecal input sources from humans and a range of animals, including cattle, pig, dog, chicken, seagull, possum, duck, and horse using quantitative PCR (qPCR) analysis of molecular marker genes^18,19^. A large number of laboratory and field studies have shown that analyses of host-associated molecular marker genes can identify sources of fecal contamination in waterways with a high degree of precision^5,20^. In addition to MST marker genes, chemical source tracking (CST) markers specific to sewage have also been used to detect sewage pollution in aquatic environments^21^. One major limitation of CST markers is that they do not consistently associate with increased risks of illness in swimmers^22^. Furthermore, little is known regarding the persistence of CST markers in aquatic environments. In contrast, substantial data are available regarding the persistence of MST marker genes in aquatic environments^19^.

Wastewater treatment plants (WWTPs) can treat human wastewater to a level that is safe for disposal in the environment or for reuse. Sewerage systems in Australia are designed with overflow (emergency relief) structures that release untreated sewage into the environmental waters when the capacity is surpassed^23^. This design is employed to minimize human-health risk from discharges within properties. A dry weather overflow occurs when there is a system failure, usually due to pipe blockages or pump failure. Wet weather overflows (WWOs) are caused by the infiltration of water into the sewage system during heavy rainfall to a point at which the hydraulic capacity of the system is exceeded. When such an event occurs, environmental and recreational waters receive sewage-contaminated stormwater, which may have an adverse impact on human and ecosystem health.

Lake Parramatta is a freshwater lake located in Sydney, NSW, which is currently used for recreational activities such as swimming, boating and water sports. The size of Lake Parramatta is approximately 10.5 ha with a designated swimming enclosure. Swimming in Lake Parramatta was officially reintroduced in January 2015. Thousands of people visit the lake for recreational activities during the summer. The swimming season usually commences in October. While there is no restriction on swimming, the local council recommends that people not swim in the lake for three days following heavy rainfall due to possible water quality deterioration.

The primary objective of this study was to determine the magnitude of sewage and animal fecal contamination in Lake Parramatta water samples collected during a dry weather period and from two storm events that coincided with WWOs. To determine the sources of sewage pollution, we used three sewage-associated marker genes [(*Bacteroides* HF183, crAssphage CPQ_056 and pepper mild mottle virus (PMMoV)]. *Bacteroides* HF183 is the most commonly used sewage-associated marker gene that belongs to the genus *Bacteroides* (an obligate anaerobe)^24^. PMMoV is the most abundant RNA virus identified in the feces of healthy individuals. PMMoV infects various species of peppers, and its presence in human feces originates from the consumption of infected peppers^25^. CrAssphage is the highly abundant DNA virus in the human gut and has been proposed for sewage pollution tracking in environmental waters^26^. Similarly, to determine the sources of animal fecal contamination, we used cattle feces-associated (CowM2), dog feces-associated (*Bacteroides* BacCan-UCD) and avian feces-associated (*Helicobacter* spp. associated GFD) marker genes. The concentrations of these DNA and RNA marker genes in water samples collected once during dry weather and compared the results with water samples collected during the two storm events. We also determined the correlations between culturable FIB and MST marker genes in lake water samples. Finally, the concentrations of HF183 and PMMoV were compared to the established marker gene threshold values that would exceed the health risk benchmark for primary contact recreators. This analysis was undertaken to indicate whether the level of sewage contamination would pose any potential risk to swimmers.

## Results

### Concentrations of fecal indicator bacteria

**Figure 1:**
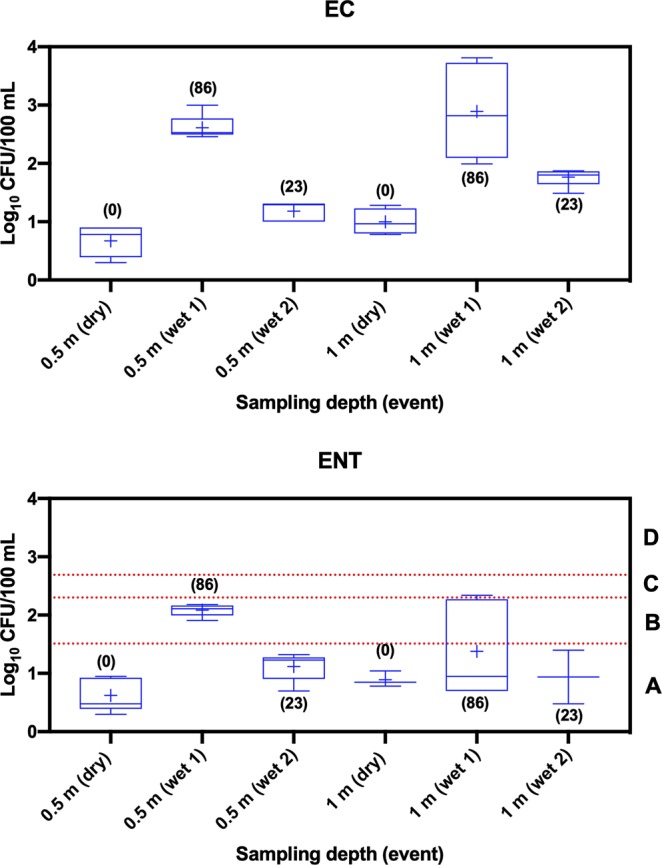
Pooled box and whisker plots of *E. coli* (EC) and *Enterococcus* spp. (ENT) in water samples collected from Lake Parramatta (LP) during dry and wet weather events. Category A (<40 CFU/100 mL), category B (41–200 CFU/100 mL), category C (201–500 CFU/100 mL) and category D (>501 CFU/100 mL). + sign represents mean concentration. Numbers in bracket show the amount of rainfall (in mm) 72 h before sampling in dry weather and 24 h before sampling of storm events.

The mean (2.62 log_10_ CFU/100 mL) concentration (pooled data from all five sites) of EC was approximately two orders of magnitude greater in samples collected during storm event 1 (study area received 86 mm prior to storm event) compared to dry weather at both sampling depths (Fig. 1). The mean concentration of EC in storm events 1 and 2 samples were significantly (*p* < 0.05) different than those samples collected in dry weather event at both sampling depths. The mean concentrations of EC at depth 0.5 m (below the water surface) did not differ significantly (*p* > 0.05) from samples that were collected at depth 1 m (above the bottom of the lake) for dry and storm event 1. However, the mean concentration of EC at 1 m was significantly different than 0.5 m below the water surface for storm event 2.

Similarly, the mean (2.08 log_10_ CFU/100 mL) concentration (pooled data from five sites) of ENT was approximately 30 times greater in samples collected during storm event 1 compared to dry weather at 0.5 m below the water surface. The mean concentration of EC in storm events 1 and 2 samples were significantly (*p* < 0.05) different from those samples collected in dry weather event at 0.5 m below the water surface but not at depth 1 m above the bottom of the lake. The concentrations of ENT in samples collected during dry weather and storm event 2 were similar, and all samples were below category A (<40 CFU/100 mL; gastrointestinal (GI) illness risk <1%) designated by NHMRC Guidelines for Recreational Use of Water^27^. In contrast, the concentrations of ENT in storm event 1 from all sites were within NHMRC B and C (>200–500 CFU/100 mL; GI illness risk 5–10%) categories at a sampling depth of 0.5 m below the water surface, while the concentration of samples from only one of five sites was above category level A at a depth of 1 m above the bottom of the lake.

### qPCR performance characteristics

qPCR standard curves (*n* = 4 to 6 separate standard curves) were analyzed to determine the qPCR performance characteristics. The standards had a linear range of quantification from 3 × 10^6^ to 3 × 10^0^ GC/reaction. The range of qPCR efficiency, linearity, slope and Y-intercept are shown in Supplementary Table S2. These parameters were within the optimal recommended value as per the MIQE guidelines^28^. The mean coefficient of variation (CV) values for all assays were <5%. The qPCR ALOD and ALOQ values were determined to range from 3 to 30 GC/reaction for all assays. No carryover contamination was observed in the method and reagent blank samples. No template controls indicated no extraneous nucleic acid contamination during the course of the study.

### Marker gene occurrence in Lake Parramatta water samples

Most (8 of 10) of the water samples collected during the dry weather event were PCR inhibitor free, as indicated by the Sketa22 assay. However, most samples that were collected during storm events (12 of 20) showed signs of PCR inhibition. A 10-fold serial dilution was made for these samples and retested with the Sketa22 assay. Reanalysis indicated that a 10-fold dilution relieved PCR inhibitory effects. Among all the sewage and animal marker genes tested, CowM2 could not be detected in any of the 30 samples collected during the dry weather and two storm events.

HF183 could not be detected in any of the samples collected during the dry weather, whereas HF183 was detected in all water samples at both depths during storm events 1 and 2 (Table 1). Similar results were also observed for CPQ_056 (DNA marker gene) and PMMoV (RNA marker gene), with increased occurrence during the storm events compared to dry weather when they were mostly absent (except one sample collected at depth 0.5 m below the water surface was positive for CPQ_056 marker gene and one sample collected at 1 m above the bottom of the lake was positive for PMMoV). The BacCan-UCD marker gene did not exhibit the same pattern and was detected throughout the three study events, however, the occurrence or detection frequency of this marker gene was much lower than the sewage-associated marker genes. The occurrence of the GFD marker at a 1 m depth was greater in storm event water samples compared to dry weather. The frequency of detection was considerably lower than sewage-associated and BacCan-UCD marker genes suggesting low levels of avian fecal pollution occurring in Lake Parramatta.

**Table 1:** Occurrence of FIB and MST marker genes in water samples collected from two depths at Lake Parramatta.

FIB and MST marker genes	No. of samples positive/No. of samples tested (%)					
	Dry weather (19/04/2018)		Storm event 1 (27/02/2018)		Storm event 2 (14/03/2018)	
	0.5 m	1 m	0.5 m	1 m	0.5 m	1 m
EC	5/5 (100)	4/5 (100)	5/5 (100)	5/5 (100)	5/5 (100)	5/5 (100)
ENT	5/5 (100)	3/5 (60)	5/5 (100)	5/5 (100)	5/5 (100)	2/5 (40)
HF183	0/5 (0)	0/5 (0)	5/5 (100)	5/5 (100)	5/5 (100)	5/5 (100)
CPQ_056	1/5 (20)	0/5 (0)	5/5 (100)	5/5 (100)	4/5 (80)	5/5 (100)
PMMoV	0/5 (0)	1/5 (20)	4/5 (80)	3/5 (60)	4/5 (80)	5/5 (100)
BacCan-UCD	2/5 (40)	1/5 (20)	2/5 (40)	4/5 (80)	0/5 (0)	1/5 (20)
CowM2	0/5 (0)	0/5 (0)	0/5 (0)	0/5 (0)	0/5 (0)	0/5 (0)
GFD	1/5 (20)	0/5 (0)	1/5 (20)	2/5 (40)	0/5 (0)	2/5 (40)

0.5 m: 0.5 m below the water surface.

1 m: 1 m above the bottom of the lake.

**Figure 2:**
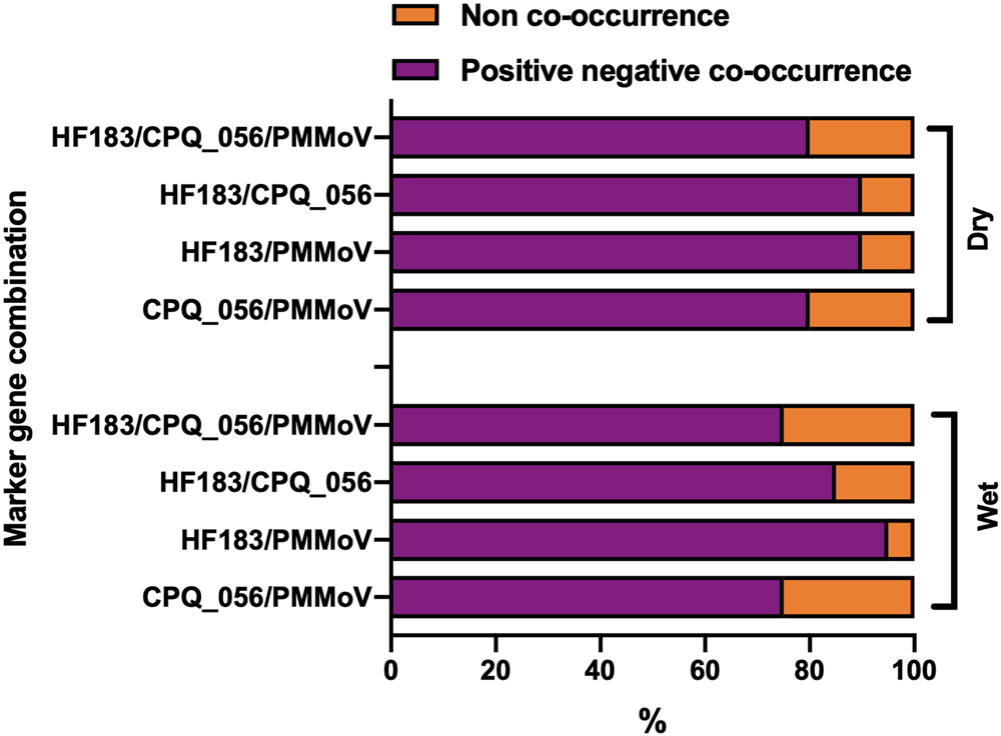
Agreement on the percentage of positive/negative co-occurrence among the *Bacteroides* HF183, crAssphage CPQ_056 and pepper mild mottle virus (PMMoV) marker genes in water samples collected from Lake Parramatta during dry and wet weather events.

Of the 20 samples collected in storm events 1 and 2, both HF183 and CPQ_056 were highly prevalent (95–100%), whereas the prevalence of the PMMoV marker gene was relatively lower (80%). The agreements (co-presence or co-absence) between the occurrence of sewage-associated marker genes in samples collected during dry and storm events were high, ranging from 80–90% (for dry weather) and 75–95% (for storm events) events (Fig. 2). During the two storm events combined, 75% of samples were positive for all three markers, whereas, 95% (HF183 and CPQ_056), 85% (HF183 and PMMoV) and 75% (CPQ_056 and PMMoV) were positive for at least two marker genes.

### Recovery efficiency

The estimated mean recovery efficiencies of the HF183 and CPQ_056 in Lake Parramatta dry weather water samples were 44.6 and 46.8%, respectively (Table 2) from three separate trials. However, the recovery efficiency of PMMoV was 20.1%, slightly lower than that of HF183 and CPQ_056. Similarly, the estimated mean recovery efficiencies of the HF183, CPQ_056 and PMMoV in Lake Parramatta wet weather water samples were 48.2, 51.2, and 28.1%, respectively.

**Table 2:** Recovery efficiency (%) of *Bacteroides* HF183, crAssphage CPQ_056 and pepper mild mottle virus (PMMoV) in water samples from Lake Parramatta.

Sample sites	Events	% recovery (mean ± SD)
HF183	Dry	44.6 ± 3.48
	Wet	48.2 ± 2.93
CPQ_056	Dry	46.8 ± 8.44
	Wet	51.2 ± 6.24
PMMoV	Dry	20.1 ± 1.19
	Wet	28.1 ± 4.32

Three separate trials were undertaken on the same water samples.

### Concentrations of sewage associated and animal-feces associated marker genes

**Figure 3:**
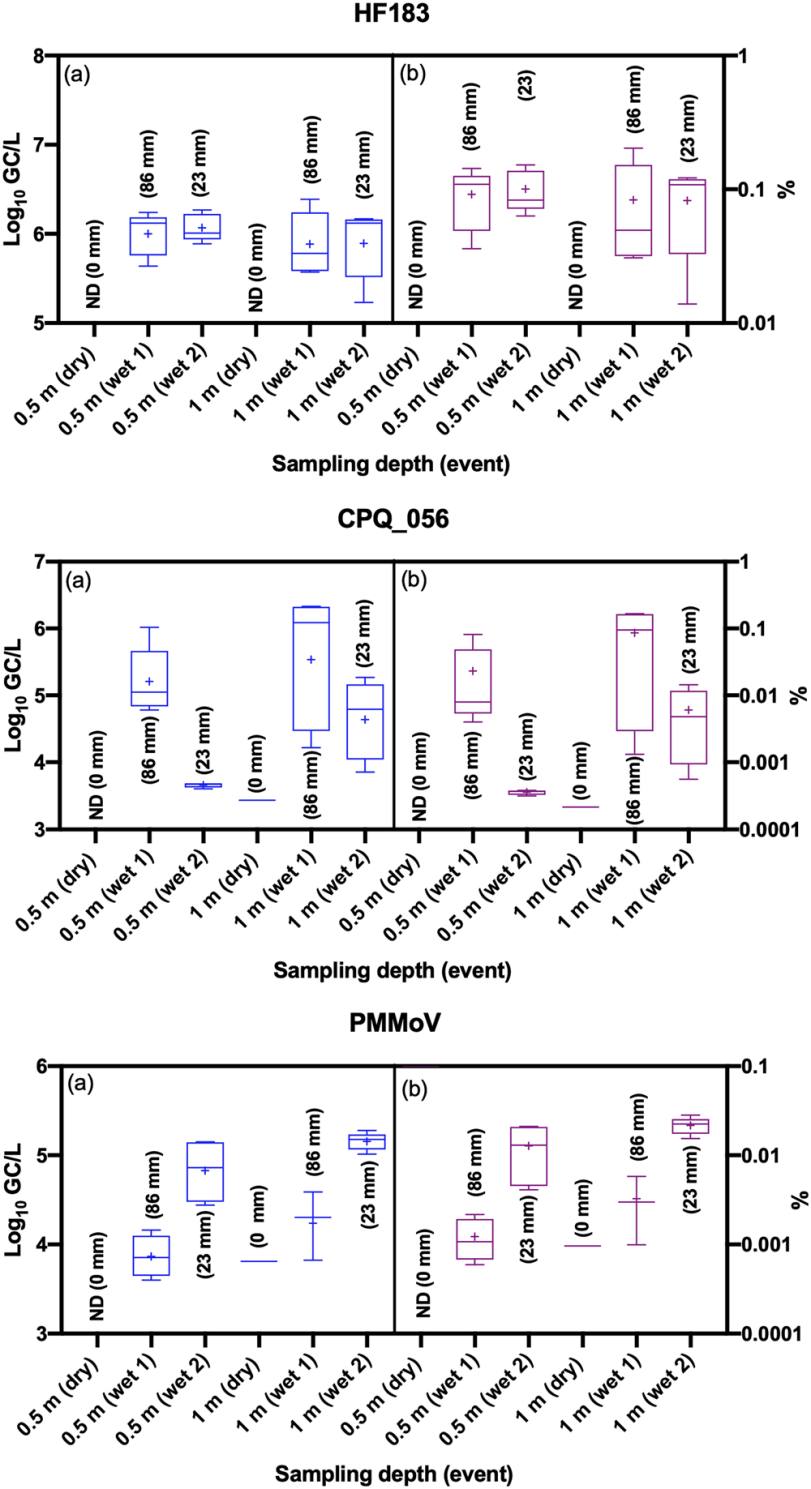
Pooled box and whisker plots of *Bacteroides* HF183, crAssphage CPQ_056 and pepper mild mottle virus (PMMoV) in water samples collected from Lake Parramatta during dry and wet weather events. Panel (a) is showing the concentrations and panel (b) is showing the percentage of sewage in water samples. + sign represents mean concentrations. Numbers in bracket showing the amount of rainfall (in mm) 72 h before sampling in dry weather and 24 h before sampling of storm events.

To estimate the concentration of sewage-associated marker genes, recovery correction was applied. The HF183 marker gene in samples collected during two storm events ranged from 5.23–6.39 log_10_ GC/L of water with a mean concentration and standard deviation of 5.96 ± 0.28 GC/L (CI 5.84–6.08) of water (Fig. 3). The mean concentrations of HF183 in samples collected from storm event 1 did not differ significantly (*p* > 0.05) from storm event 2 at either sampling depth. Additionally, the mean concentrations of the HF183 marker did not differ significantly (*p* > 0.05) between the depths during both storm events.

The CPQ_056 marker gene in samples collected during storm events ranged from 3.61–6.33 log_10_ GC/L of water with a mean concentration and standard deviation of 4.82 ± 0.91 GC/L (CI 4.42–5.22) of water. Only one sample collected during the dry weather at a depth 0.5 m below the water surface was positive for CPQ_056, and the concentration was 3.44 log_10_ GC/L. The mean concentration of HF183 in storm event 1 was significantly (*p* < 0.05) different than the storm event 2 at a depth of 0.5 m below the water surface, but the concentrations did not differ considerably (*p* > 0.05) between storm events 1 and 2 at a depth of 1 m above the lake bottom surface. Additionally, the mean concentrations of the HF183 marker did not differ significantly (*p* > 0.05) between depths during storm event 1, but a statistically significant (*p* < 0.05) difference was shown for event 2 between the two depths.

The PMMoV marker gene in samples collected during both storm events ranged from 3.60–5.27 log_10_ GC/L of water with a mean concentration and standard deviation of 4.58 ± 0.58 GC/L (CI 4.30–4.86) of water. Only one sample collected during the dry weather at a depth of 1 m above the lake bottom surface was positive for the PMMoV, and the concentration was 3.81 log_10_ GC/L. The mean concentration of PMMoV in storm event 1 was significantly (*p* < 0.05) different than the storm event 2 at both depths, while the mean concentrations of the PMMoV marker did not differ significantly (*p* > 0.05) between depths during storm events 1 and 2.

**Figure 4:**
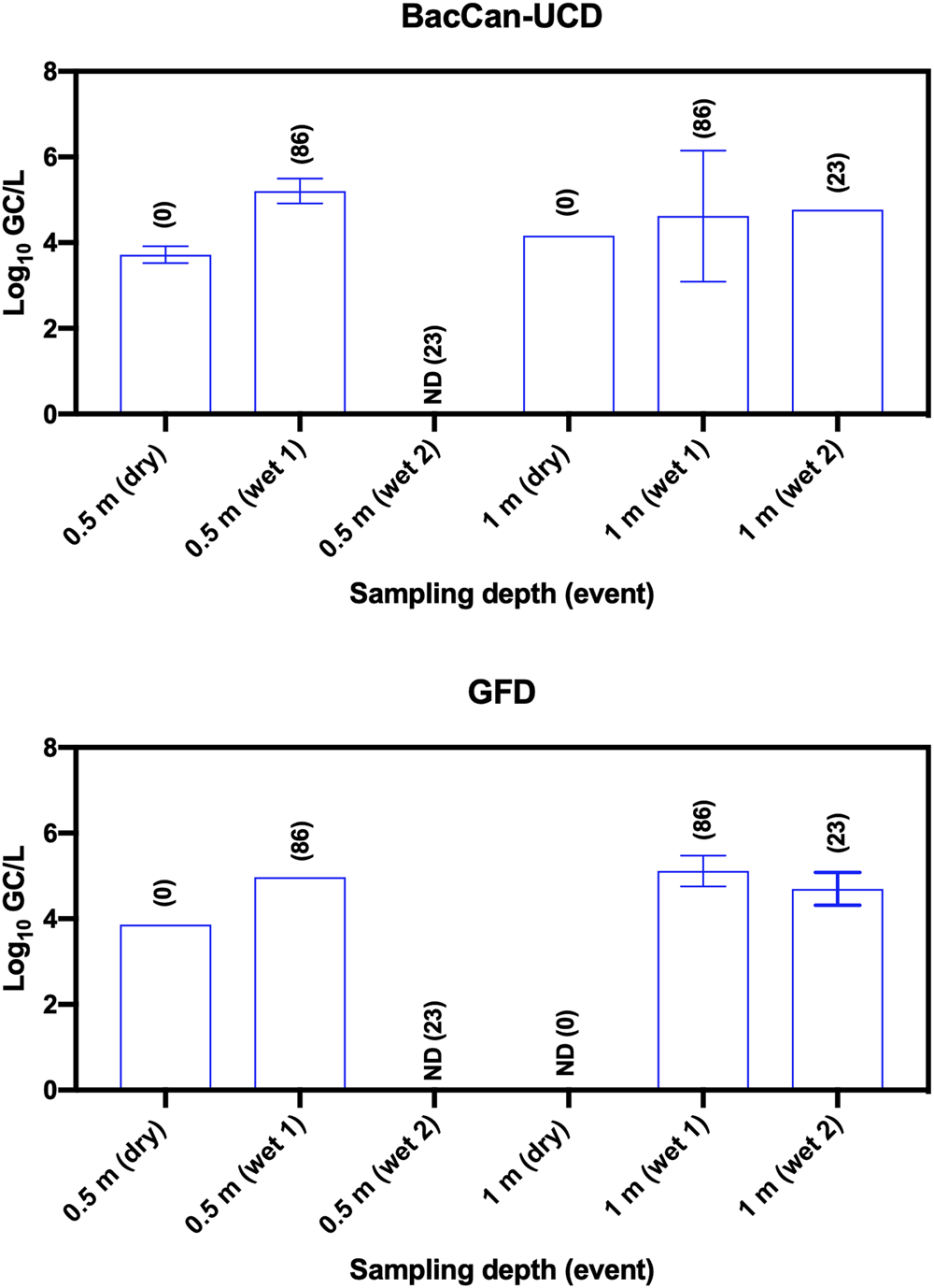
Concentrations of *Bacteroides* BacCan-UCD and *Helicobacter* spp. associated GFD marker genes in water samples collected from Lake Parramatta during dry and wet weather events. Numbers in bracket show the amount of rainfall (in mm) 72 h before sampling in dry weather and 24 h before sampling of storm events.

BacCan-UCD, cowM2 and GFD marker gene recovery efficiencies were not determined in this study. The BacCan-UCD marker gene in PCR quantifiable samples collected during dry weather events ranged from 3.38–3.56 log_10_ GC/L of water with a mean concentration and standard deviation of 3.47 ± 0.09 GC/L (CI 3.37–3.57) of water (Fig. 4). The BacCan-UCD marker gene in PCR quantifiable samples collected during storm events ranged from 4.52–5.47 log_10_ GC/L of water with a mean concentration and standard deviation of 4.90 ± 0.34 GC/L (CI 4.65–5.15) of water. The mean concentrations of BacCan-UCD in dry weather samples were similar to those of storm event samples. Statistical analysis could not be performed to identify significant differences in the concentration of BacCan-UCD between storm events and depths due to the small numbers (10/30 in total) of PCR quantifiable samples.

The GFD marker gene in PCR quantifiable samples collected during both storm events ranged from 4.09–5.03 log_10_ GC/L of water with a mean concentration and standard deviation of 4.58 ± 0.33 GC/L (CI 4.12–5.04) of water. The GFD marker gene in one and only PCR quantifiable samples collected during dry weather event was 3.52 log_10_ GC/L. Statistical analysis could not be performed to identify significant differences in the concentration of GFD between storm events and depths due to the small numbers (6/30 in total) of PCR quantifiable samples.

### Correlations among FIB and MST marker genes

**Figure 5:**
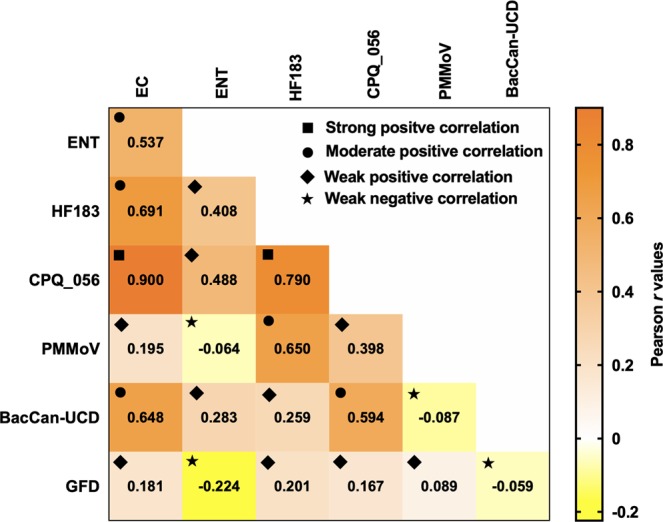
Pearson’s correlation matrix among fecal indicator bacteria (FIB) and MST marker genes in pooled (*n* = 30) water samples collected from Lake Parramatta during dry and wet weather events. EC: *E. coli*; ENT: Enterococci.

Since FIB are used for regulatory purposes, from a public health perspective, the relationship between FIB and MST markers is important. In view of this, we determined the relationship among FIB and MST marker genes tested in this study. A significant (*p* < 0.05) positive moderate correlation (*r* = > 0.5 but <0.7) was observed between culturable EC and ENT (Fig. 5). A significant (*p* < 0.05) strong positive correlation (*r* = > 0.7) was also found between EC and CPQ_056, while EC was also significantly (*p* < 0.05) and moderately correlated with both HF183 and BacCan-UCD. Although EC showed a weak positive correlation with PMMoV and GFD, the relationship was not statistically significant (*p* > 0.05). Positive moderate significant (*p* < 0.05) correlations were observed between ENT vs. HF183 and ENT vs. CPQ_056. ENT negatively correlated with PMMoV and GFD. HF183 and CPQ_056 showed a strong significant (*p* < 0.05) positive correlation (*r* = 0.79), suggesting a high association between these two marker genes in Lake Parramatta pooled water samples. HF183 and PMMoV moderately correlated with each other. A significant (*p* < 0.05) moderate correlation was also found between PMMoV and CPQ_056. In general, the GFD marker gene showed either a weak correlation or negative correlation with FIB and other sewage-associated markers. *p* values for the FIB and MST marker gene associations are shown in supplementary Table S3.

### Comparison of concentrations of marker genes to GI illness risk benchmark

Research studies have established relationships between concentrations of sewage-associated marker genes such as HF183 and PMMoV to GI risk of swimmers in recreational waters contaminated with fresh untreated sewage using a quantitative microbial risk assessment (QMRA)^29–31^. The concentration of PMMoV in one sample that was collected during the dry weather event did not exceed the risk benchmark value but the rest of the samples collected during the storm events did (Table 3). The concentrations of the HF183 in all quantifiable samples exceeded the established GI risk benchmarks for 30 or 36 GI illness/1,000 primary contact recreators for a swimming event, suggesting that swimming immediately after the storm event may pose a human health risk.

**Table 3:** Number of samples that exceeded the Gastrointestinal (GI) illness risk threshold for *Bacteroides* HF183 and pepper mild mottle virus (PMMoV) marker genes in water samples collected from Lake Parramatta.

Sewage-associated marker genes	GI illness risk benchmark threshold (log_10_ GC/100 mL)	No. of samples exceeded GI illness risk benchmark/No. of samples tested (%)					
		Dry weather (19/04/2018)		Storm event 1 (27/02/2018)		Storm event 2 (14/03/2018)	
		0.5 m	1 m	0.5 m	1 m	0.5 m	1 m
HF183	3.50^*a*^ 3.62^*b*^ 3.98^*c*^	0/5 (0) 0/5 (0) 0/5 (0)	0/5 (0) 0/5 (0) 0/5 (0)	5/5 (100) 5/5 (100) 5/5 (100)	5/5 (100) 5/5 (100) 5/5 (100)	5/5 (100) 5/5 (100) 5/5 (100)	5/5 (100) 5/5 (100) 5/5 (100)
PMMoV	2.73^*a*^	0/5 (0)	1/5 (20)	4/5 (80)	3/5 (60)	4/5 (80)	5/5 (100)

0.5 m: 0.5 m below the water surface; 1 m: 1 m above the bottom of the lake; ^*a*^:^30^; ^*b*^:^29^; ^*c*^:^31^.

## Discussion

Concentrations of both culturable FIB in lake water samples during the dry weather event suggested the occurrence of low levels of fecal pollution. This low leveal of pollution is also supported by the absence of sewage-associated marker genes in most of the dry weather samples. These results suggest that FIB in lake water samples did not originate from sewage during the dry weather event. However, a small number of samples were positive for BacCan-UCD (dog) and GFD (avian) marker genes, suggesting the occasional presence of dog and avian fecal pollution, and these are the likely sources of FIB observed in lake water samples in the dry weather event. Our findings corroborate a previous study that showed that wildlife might have contributed ENT in Lake Parramatta during dry weather event^32^. The mean concentration of EC in storm event 1 was approximately two orders of magnitude greater than in dry weather. However, storm event 2 did not exhibit such a pattern. There was a slight increase in EC concentration in storm event 2 compared to dry weather. This increase may be due to rainfall intensity as during storm event 1, Lake Parramatta received 78 mm rainfall compared to storm event 2 when the study area received 23 mm rainfall. ENT also showed similar trends as EC with increased concentrations after storm event 1 compared to dry weather and storm event 2.

To determine the impacts of WWOs on Lake Parramatta, we used three sewage-associated molecular marker genes. The rationale for using three marker genes is that these marker genes are highly abundant in untreated sewage; therefore, they allow detection of a minute amount of diluted sewage in environmental water samples. We also determined the recovery efficiencies of sewage-associated marker genes in water samples collected from Lake Parramatta. Recovery efficiencies were not determined for lower priority animal feces-associated marker genes because sewage is the primary source of interest from a public health perspective in the study lake. For the determination of recovery efficiencies, lake water samples were seeded with untreated sewage, rather than a single sample processing control (i.e., a bacterium or a virus). This step was undertaken to obtain information on the recovery efficiencies of the actual marker genes rather than an SPC whose recovery efficiency may be different from the marker genes tested in this study. The recovery efficiencies of marker genes were similar in Lake Parramatta water samples collected during the dry and wet weather events, and the values were similar to those obtained in our previous study^33^.

We also evaluated the host specificity of these marker genes in our previous studies^34,35^. Both HF183 and CPQ_056 marker genes were found to be highly associated with sewage. Although none of these MST markers shows absolute host specificity, they can be occasionally found in nonhuman hosts. For example, in a recent study^34^, we reported low levels of the crAssphage CPQ_056 marker gene in chicken and cattle wastewater samples. False positive source identification (i.e., when a source is absent, the analysis is showing it is present in water) is problematic because it may lead to wasted capital investment to rectify the problem. When a water sample is simultaneously positive for two or three sewage-associated marker genes, it increases the confidence level that the source of the contamination has been identified correctly.

In this study, sewage-associated marker genes could not be detected in dry weather samples when no gauged overflows occurred; however, most of the wet weather samples from both storm events were positive for HF183 (i.e., bacterial DNA marker gene), crAssphage (i.e., viral DNA marker gene) and PMMoV (i.e., viral RNA marker gene) marker genes, suggesting sewage pollution is occurring in the lake due to WWOs, which had recorded gauged discharges for both storm events. A previous study undertaken in the same lake approximately a decade ago also reported notably high coprostanol concentrations (14,000 ng/L) in stormwater^32^. The levels of HF183, crAssphage and PMMoV in water samples collected during storm events were as high as 6.43, 6.37 and 5.42 log_10_ GC/L of water, respectively. These levels are somewhat expected in stormwater/WWOs samples and have been reported in other studies in urban environments originating from sewage-contaminated stormwater^36–38^.

Among the three sewage-associated marker genes tested, HF183 and crAssphage were more prevalent in water samples than the PMMoV markers. This finding is observed because the abundance of marker genes varies in untreated sewage depending on their prevalence in the human host. For example, the concentration of the HF183 marker gene is one- to two-orders of magnitude greater in untreated sewage compared to viral marker genes such as human adenovirus (HAdV), human polyomavirus (HPyV) or PMMoV^39^. As a result, HF183 may be detected more frequently in environmental waters compared to HAdV and HPyV or PMMoV. In addition, the decay of DNA marker and RNA marker genes may be different in the environment affecting their detection. Nonetheless, during storm events, 75% of the samples were positive for all three markers, whereas 75–95% of the samples were positive for at least two marker genes, suggesting good agreement among these markers to identify sewage pollution in the studied lake. A strong positive significant correlation was observed between HF183 and CPQ_056, while PMMoV was moderately and significantly correlated with HF183 and CPQ_056.

It can be difficult to interpret the marker gene abundance data in recreational water samples in the context of human health risk. This information is crucial to catchment managers to formulate effective risk mitigation strategies. In recent years, studies^29,30,40^ have established a link between the abundance of marker genes and human health risk using the US EPA Recreational Water Quality Criteria (RWQC) benchmark value for primary contact recreation^41^. For example, Boehm and colleagues^29^ reported that a median gastrointestinal (GI) illness rate of 30/1,000 primary contact recreators occurred when the median concentration of HF183 in a hypothetical waterbody contaminated with fresh untreated sewage was 3.62 log_10_ GC/100 mL. In a recent study, the same authors improved their earlier marker threshold value estimation by incorporating the decay of HF183 and reference pathogens^31^. For a fresh untreated sewage contamination event in a hypothetical waterbody, the HF183 marker gene threshold was ~3.98 log_10_ GC/100 mL to exceed the primary contact GI illness rate^31^. Ahmed and colleagues^30^ reported that a median concentration of 3.50 and 2.73 log_10_ GC/100 mL of HF183 and PMMoV in untreated sewage contaminated recreational water represented a risk above the illness benchmark of 36/1,000 primary contact recreators.

Since the concentrations of the HF183 and PMMoV marker genes were greater in storm event samples, we compared the abundance of these marker genes to the values established above to determine the number of samples that exceeded the RWQC illness benchmark values. All samples collected during the wet weather events exceeded the risk benchmark value for primary recreation, suggesting that swimming in Lake Parramatta including the designated swimming area (i.e., LP02A) after storm events will increase the risk of GI illness above the benchmark value. However, sewage pollution will age in Lake Parramatta if there are no further WWOs. Moreover, environmental stressors such as temperature, sunlight, UV, predation and environmental matrix, will influence the decay of both sewage-associated markers and reference pathogens^10,42–46^.

Boehm and colleagues^31^ estimated that when sewage contamination in surface waters is >3.3 days old, exposure to sewage will be unlikely to result in GI illness risk greater than 30/1,000 primary contact recreators due to the decay of both HF183 and reference pathogen norovirus. A recent study also showed that the indigenous bacterial community of water samples collected from a freshwater lake recovered and accounted for approximately 70% of the baseline community within 4 days following sewage addition^47^. The Beachwatch water quality monitoring program in Sydney, NSW routinely monitors beach water quality throughout the swimming season by measuring ENT. This monitoring is based on the NHMRC guideline^27^. The NSW Office of Environment and Heritage recommends avoiding swimming at ocean beaches at least one day and after heavy rain and for up to three days at estuarine swimming waters.

It is highly likely that the concentrations of ENT in Lake Parramatta would reach a safe level (i.e., category A) due to the rapid decay of ENT within 3–4 days after a rainfall event^48,49^. However, pathogens such as enteric viruses and protozoa may persist longer in the environmental waters due to their high stability during environmental stressors^49,50^. Therefore, the decay rates of sewage-associated marker genes and reference pathogens need to be incorporated into the dose-response models for accurate risk estimates and to determine when sewage-contaminated water is safe for swimming. However, in this study, we compared the MST marker gene abundance with existing marker gene threshold values developed in Southeast, Queensland, Australia and California, USA. These studies used location specific or metadata to estimate the norovirus dose. The concentrations of norovirus are generally higher in the winter season (nonswimming period) than in the summer season and vary substantially in untreated sewage^51^. This variation needs to be considered when interpreting MST marker gene abundance data, as this will generate different marker gene threshold values.

Among the animal feces-associated marker genes tested, cowM2 could not be detected in water samples suggesting that cow fecal pollution had not occurred throughout the study period. Cattle are known to harbor a variety of pathogens such as *E. coli* O157:H7, *C. parvum*, *C. jeuni*, and *Salmonella* spp.^8,52^. Based on the results, we can conclude that the risk from cattle-borne pathogens is low in the studied lake. Dog- and avian-feces-associated markers were sporadically detected, although the concentrations were not high enough to pose any risk to human health. There is no marker gene threshold available for BacCan-UCD and GFD marker genes. However, Brown and colleagues^53^ determined that the median concentration of illness equals the GI risk benchmark when gull feces associated *Catellicoccus* marker gene concentration is 6.60 log_10_ GC/100 mL. In this study, the avian marker concentrations in water samples were well below the required concentrations to exceed the risk benchmark.

In conclusion, the results from multiple sewage-associated marker gene analysis clearly demonstrate that the study lake is impacted by sewage contamination following storm events that deliver WWOs. Ongoing advice to not swim for several days after storm events appears to be appropriate. Low levels of fecal pollution and sewage contamination (mostly absent) during the dry weather event suggested that swimming in Lake Parramatta may not pose human health risks. This finding is also in accordance with low FIB abundance in lake water samples. Taken together, these results suggest that testing more dry events in water samples may not be necessary. Further research is required to obtain insight into the decay rates of sewage-associated marker genes in relation to each other and enteric viruses. That research would accurately establish what levels of markers would pose a human health risk to swimmers and whether the period to avoid swimming can be better defined to minimize potential health risk. The risk from exposure to pathogens, especially enteric viruses, remains unknown and warrants further study along with currently used FIB to assess their suitability for lake water monitoring to better understand illness-risk from WWOs and storm events without WWOs. The MST tools used in this study can enhance our understanding of contamination sources and aid in management understanding to help guide the implementation of best management practices and remediation efforts to improve bathing water quality and diminish human-health illness-risk.

## Materials and Methods

### Water sampling

Five sampling sites (LP1, LP2, LP02A LP3, and LP5) were chosen from Lake Parramatta (see Supplementary Fig. S1). Among the five sampling sites, only LP02A is located within the designated swimming enclosure, although people swim in other parts of the lake at their own discretion. At all five sites, samples were collected at two depths: (i) 0.5 m below the water surface, and (ii) 1 m above the bottom of the lake. Water samples were collected during dry weather (19/4/2018) and two storm events (27/2/2018 and 14/3/2018) with gauged sewer overflows yielding a total of 30 water samples.

For this study, a dry weather event is represented by no precipitation for at least 72 h prior to sampling. The storm weather event was represented by a minimum of 10 mm of precipitation in the preceding 24 h. The study area received 86 mm and 23 mm rainfall on storm events 1 and 2, respectively. To understand when sewage discharges occur to the Lake Parramatta catchment, level sensors with telemetry were placed at four designated overflow points. All four overflow points enter the catchment above the uppermost lake sampling site of LP5. The lowest of the four overflows discharged on 25/02/2018 for 18 h and 35 min and on 26/02/2018 for 50 min. While approximately midnight 13/03/2018 an overflow of 40 min duration was also recorded. Hence in this study, WWOs were a mixture of urban stormwater and diluted sewage. A 5 L sample was collected from each occasion using a sterile plastic container. Samples were transported to the laboratory on ice and processed within 6 h of collection.

### FIB enumeration

FIB enumeration was conducted at the Sydney Water laboratory. Colilert^®^ (IDEXX Laboratories, Westbrook, Maine, USA) was used to determine the concentrations of EC in 10 mL and 100 mL of each water sample. Heat-sealed Quanti trays were incubated at 35 ± 0.5 °C for 18 h but no longer than 22 h as per the manufacturer’s recommendation. The most probable number (MPN) of EC present was determined by the total number of yellow/fluorescent wells. The membrane filtration method was used for ENT enumeration. Briefly, 10 and 100 mL of water samples were filtered through 0.45 µm cellulose ester membranes (Millipore, Japan). The membrane was then placed onto m *Enterococcus* agar plates (Edwards, Australia) and incubated at 36 ± 2 °C for 44 ± 4 h. To confirm ENT, the membrane was transferred onto preheated Enterococcosel Agar (ECSA) plates (Edwards, Australia) at 44 °C and incubated for 2 h at 44 ± 0.5 °C. Colonies showing a tan to black/brown color in the surrounding medium were enumerated as ENT.

### Processing of water samples

The pH in all water samples was adjusted to 3.5 using 2.0 N HCl, and approximately 500 mL -2 L of each sample was filtered in duplicate (one set for DNA analysis and the other set for RNA analysis) through negatively charged 47-mm or 90-mm, 0.45-μm HA membranes (Millipore) to capture both bacteria and viruses^33,34^. In the case of membrane clogging due to turbidity, multiple membranes were used. The membranes were inserted into 15 mL tubes and stored at −80 °C for 1 month. The membranes were shipped to the CSIRO Laboratory in Brisbane for DNA extraction and qPCR analysis.

### Nucleic acid extraction and PCR inhibition testing

Upon arrival, DNA and RNA samples were separately extracted from the duplicate membranes using the DNeasy and RNeasy Power Water Kits (Qiagen, Valencia, CA). Extracted nucleic acid concentrations were measured with a spectrophotometer (NanoDrop ND-1000, Thermo Scientific, Willmington, DE, USA). cDNA was synthesized immediately after RNA extraction using the SuperScript™ VILO™ cDNA Synthesis Kit (Invitrogen, Carlsbad, CA, USA) according to the manufacturer’s instructions. An 8 µL aliquot of extracted RNA was used to generate 20 µL of cDNA.

An experiment was conducted to determine the presence of PCR inhibitors in environmental DNA/cDNA samples using a Sketa22 real-time PCR assay^54^. Water samples with a two quantification cycle (Cq) delay were considered to have PCR inhibitors^40^. Samples with PCR inhibitors were subjected to a 10-fold dilution with TE buffer. Undiluted (i.e., PCR uninhibited) and 10-fold diluted (i.e., PCR inhibited) samples were used for qPCR analysis of marker genes. All DNA samples were stored at −80 °C and subjected to qPCR analysis within five to seven days after nucleic acid extraction.

### Recovery efficiency

Since sewage contamination is important from a public health perspective in the study lake, recovery efficiencies were determined to determine the actual concentrations of HF183, CPQ_056, and PMMoV. To determine the recovery efficiency of the concentration method, raw sewage samples were collected from a WWTP in Sydney, Australia. DNA and RNA were directly extracted from 500 µL of raw sewage using DNeasy and RNeasy Power Water Kits in triplicate (Qiagen, Valencia, CA). The concentrations of HF183, CPQ_056 and PMMoV in sewage DNA samples were determined using qPCR assays (see below for methodological details).

Water samples collected from Lake Parramatta during dry weather and storm weather events were seeded with raw sewage. In brief, 50 mL of sewage sample was added to 950 mL of Lake Parramatta water samples in triplicate. Three separate trials were undertaken. Viruses and bacteria were concentrated from sewage seeded water samples using the methods described earlier. After filtration, each membrane was cut into half. DNA and RNA were directly extracted from each half of the membrane using DNeasy and RNeasy Power Water Kits in triplicate (Qiagen, Valencia, CA). The recovery efficiencies of HF183, CPQ_056, and PMMoV for sewage seeded Lake Parramatta samples were determined as follows: recovery efficiency (%) = (concentration of sewage-associated marker gene recovered/concentration seeded) × 100.

### qPCR assays

Previously published qPCR assays were used for the analysis of the HF183, crAssphage CPQ_056, PMMoV, BacCan-UCD, cowM2 and GFD marker genes^24,26,55–58^. The primers and probes for these qPCR assays are shown in Supplementary Table S1 along with qPCR cycling parameters. For all qPCR assays, synthetic DNA fragments in plasmid cloning vectors, containing 132 bp (for HF183), 125 bp (for CPQ_056), 68 bp (for PMMoV), 145 bp (for BacCan-UCD), 92 bp (for cowM2) and 123 bp (for GFD) were purchased from Integrated DNA Technologies (Coralville, IA, USA).

qPCR standards were prepared from the synthetic DNA, ranging from 10^6^ to 1 GC/µL of DNA. HF183, CPQ_056, PMMoV, BacCan-UCD, and cowM2 qPCR amplifications were performed in 20 µL reaction mixtures using SsoAdvanced Universal Probes Supermix (Bio-Rad Laboratories, Richmond, CA). HF183, CPQ_056, and cowM2 qPCR mixtures contained 10 µL of Supermix, 1000 nM of forward primer, 1000 nM of reverse primer, 100 nM probe and 3 µL of template DNA. PMMoV qPCR mixture contained 10 µL of Supermix, 200 nM of forward primer, 200 nM of reverse primer, 80 nM probe and 3 µL of template DNA. BacCan-UCD qPCR mixture contained 10 µL of Supermix, 400 nM of forward primer, 400 nM of reverse primer, 100 nM probe and 3 µL of template DNA.

GFD qPCR amplifications were performed in 20 µL reaction mixtures using SsoAdvanced Universal SYBR Green Supermix (Bio-Rad Laboratories, Richmond, CA). GFD mixture contained 10 µL of Supermix, 100 nM of forward primer, 100 nM of reverse primer and 3 µL of template DNA. To separate the specific product from non-specific products, including primer dimers, a melting curve analysis was performed for each qPCR run. During melt curve analysis, the temperature was increased from 65 to 95 °C at 0.5 °C increment. Melting curve analysis showed a distinct peak at a temperature of 84 ± 0.2 °C, indicating a positive and correct amplification. The qPCR assays were performed using a Bio-Rad CFX96 thermal cycler. All qPCR reactions were performed in triplicate. For each qPCR run, a series of standards (3 × 10^6^ to 3 GC/reaction) and no template controls (*n* = 3) were included.

### qPCR performance characteristics

qPCR standards were analyzed to determine the amplification efficiencies (E) and the correlation coefficient (*R*^2^). The reproducibility of the HF183, CPQ_056 and PMMoV assays were assessed by determining inter-assay reproducibility. Inter-assay variability was measured using six separate qPCR assays performed on four different days using three replicates of the standard ranging from 3 × 10^6^–3 × 10^1^ GC/reaction. The qPCR assay’s lower limit of detection (ALOD) values were determined from Cq values of the multiple standards run as described elsewhere^40^.

### Quality control

Field and method blanks were used to ensure that no carryover contamination occurred between sampling events. In addition, a reagent blank was included for each batch of DNA samples to ensure no carryover contamination occurred from DNA extraction reagents. To minimize qPCR contamination, nucleic acid extraction and qPCR setup were performed in separate laboratories.

### Data analysis

For statistical analysis, sewage-associated marker genes concentrations were log_10_ transformed. For correlation analysis, all samples collected during the study were pooled. The Pearson’s product moment correlation with a two-tailed *p* value was used to establish the relationship between FIB and MST marker genes in Lake Parramatta water samples. In general, *r* = >0.7 was considered a strong positive correlation, *r* = >0.5 but <0.7 was moderate positive correlation, and *r* = >0.1 but <0.5 was weak positive correlation. GraphPad Prism 8 was used for statistical analysis (GraphPad Software, Inc.). FIB and marker gene concentrations were assumed to be one-half of the ALOD when they were not detected^59,60^. A student’s t-test was used to identify significant relationships of the concentrations of FIB and MST marker genes between dry and wet weather events and between sampling depths.

## Supplementary information

Supplementary materials
