# Supplementary material for: Enhanced insights from human and animal host-associated molecular marker genes in a freshwater lake receiving wet weather overflows
Source: Sci Rep. 2019 Aug 29;9:12503. doi: 10.1038/s41598-019-48682-4 (PMC6715810; doi:10.1038/s41598-019-48682-4)
Supplement: Supplementary file 1 — Supplementary materials [file 41598_2019_48682_MOESM1_ESM.pdf]

Enhanced Insights from human and animal host-associated molecular marker genes in a freshwater lake receiving wet weather overflows

Warish Ahmed[1,*], Sudhi Payyappat[2], Michele Cassidy[2], and Colin Besley[2]

[1]CSIRO Land and Water, Ecosciences Precinct, 41 Boggo Road, Dutton Park, QLD 4102, Australia

[2]Sydney Water, 1 Smith Street, Parramatta, NSW 2150, Australia

Running title: Sewage pollution of inland waters

Corresponding author. Warish Ahmed. Mailing address: Ecosciences Precinct, 41 Boggo Road, Dutton Park 4102, Queensland, Australia. Tel.: +617 3833 5582; E-mail address: Warish.Ahmed@csiro.au

Supplementary Materials

Supplementary Table S1:
Primers and probes used in this study

Assays	Primers and probes	Primer and probe concentration	Cycling parameters	References
Sketa22	F:GGT TTC CGC AGC TGG G	500 nM	10 min at 95°C, 40 cycles of	1
	R:CCG AGC CGT CCT GGT CTA	500 nM	15 s at 95°C, 45 s at 63°C	
	P:FAM-AGT CGC AGG CGG CCA CCG T-TAMRA	400 nM		
Bacteroides	F:ATC ATG AGT TCA CAT GTC CG	1000 nM	10 min at 95°C, 40 cycles of	6
HF183	R:CTT CCT CTC AGA ACC CCT ATC C	1000 nM	15 s at 95°C, 60 s at 60°C	
	P:FAM-CTA ATG GAA CGC ATC CC-BHQ-1	100 nM		
CrAssphage	F:CAG AAG TAC AAA CTC CTA AAA AAC GTA GAG	1000 nM	10 min at 95°C, 40 cycles of	7
CPQ_056	R:GAT GAC CAA TAA ACA AGC CAT TAG C	1000 nM	15 s at 95°C, 60 s at 60°C	
	P:Quasar 670-AAT AAC GAT TTA CGT GAT GTA AC-BHQ-3	100 nM		
PMMoV	F: GAG TGG TTT GAC CTT AAC GTT TGA	200 nM	10 min at 95°C, 45 cycles of	4
	R:TTG TCG GTT GCA ATG CAA GT	200 nM	30 s at 95°C, 60 s at 53°C,	
	P:FAM-CCT ACC GAA GCA AAT G-MGBNFQ	80 nM	60 s at 72°C, 10 min at 72°C	
BacCan-UCD	F:GGA GCG CAG ACG GGT TTT	400 nM	10 min at 95°C, 40 cycles of	2
	R:CAA TCG GAA TTC TTC GTG ATA TCT A	400 nM	15 s at 95°C, 60 s at 60°C	
	P:FAM-TGG TGT AGC GGT GAA A-TAMRA	100 nM		
CowM2	F:CGG CCA AAT ACT CCT GAT CGT	1000 nM	10 min at 95°C, 40 cycles of	3
	R:GCT TGT TGC GTT CCT TGA GAT AAT	1000 nM	15 s at 95°C, 60 s at 60°C	
	P:FAM-AGG CAC CTA TGT CCT TTA CCT CAT CAA CTA CAG ACA-TAMRA	80 nM		
GFD	F:TCG GCT GAG CAC TCT AGG G	100 nM	10 min at 95°C, 45 cycles of	5
	R:GCG TCT CTT TGT ACA TCC CA	100 nM	10 s at 95°C, 30 s at 57°C, 20 s at 72°C	

Supplementary Table S2:
qPCR assay performance characteristics

Performance characteristics	HF183	CPQ_056	PMMoV	BacCan-UCD	CowM2	GFD
Efficiency (E) (%) (range)	97.3 to 102	96.5 to 107	94.3 to 98.2	91.2 to 93.4	96.2-104	98.2-99.3
Linearity (R^2) (range)	0.998 to 0.999	0.973 to 0.999	0.978 to 0.999	0.988 to 0.999	0.996 to 0.999	0.995 to 0.999
Slope (range)	-3.482 to -3.242	-3.412 to -3.154	-3.510 to -3.258	-3.412 to -3.154	-3.456 to -3.345	-3.387 to -3.245
Y-intercept (range)	38.12 to 39.23	37.81 to 39.86	37.92 to 38.80	38.29 to 40.02	38.56 to 39.90	38.83 to 40.10
Inter CV (%) (range)	2.33 to 4.95	4.44 to 7.19	3.32 to 4.44	2.53 to 4.95	4.10 to 6.12	1.33 to 3.22

Efficiency (E) = $10^{(1/-slope)}$; CV = coefficient of variation

Supplementary Table S3:

P values for Pearson's product moment correlation analysis among fecal indicator bacteria and MST marker genes

	EC	ENT	HF183	CPQ_05	PMMoV	BacCan-UCD
ENT	**0.0022**					
HF183	**<0.0001**	**0.0252**				
CPQ_056	**<0.0001**	**0.0062**	**<0.0001**			
PMMoV	0.3024	0.7377	**0.0001**	**0.0294**		
BacCan-UCD	**0.0001**	0.1295	0.1675	**0.0005**	0.6461	
GFD	0.3391	0.2345	0.2862	0.3770	0.6389	0.7580

Bold-faced values are statistically significant.

Supplementary Fig. S1:
Map showing sampling sites located in three estuarine and a freshwater lake in Sydney.

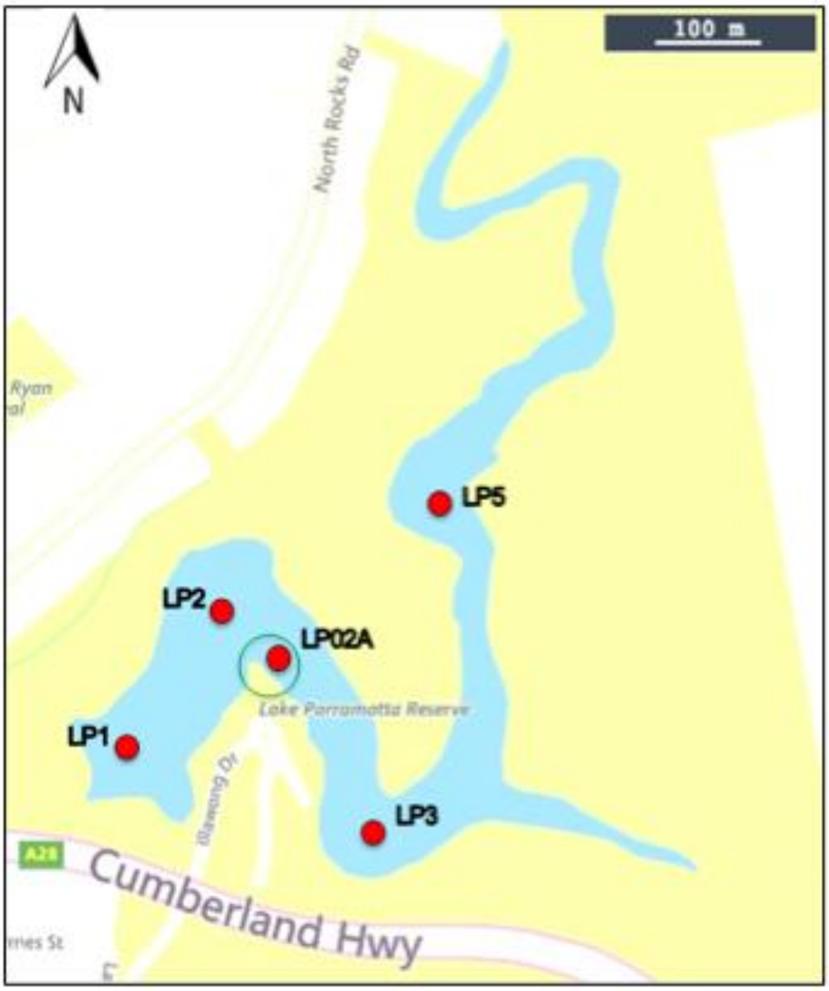

References

1. Haugland, R.A., Siefring, S.C., Wymer, L.J., Brenner, K.P., & Dufour, A.P. Comparison of *Enterococcus* measurements in freshwater at two recreational beaches by quantitative polymerase chain reaction and membrane filter culture analysis. *Water Res.* **39,** 559-568 (2005).
2. Kildare, B.J,, Leutenegger, C.M., McSwain, B.S., Bambic, D.G., Rajal, V.B., & Wuertz S. 16S rRNA-based assays for quantitative detection of universal, human-, cow-, and dog-specific fecal Bacteroidales: a Bayesian approach. *Water Res.* **41,** 3701-3715 (2007).
3. Shanks, O.C., Atikovic, E., Blackwood, A.D., Lu, J., Noble, R.T., Santo Domingo, J., Seifring, S., Sivaganesan, M., & Haugland, R.A. Quantitative PCR for detection and enumeration of genetic markers of bovine fecal pollution. *Appl. Environ. Microbiol.* **74,** 745-752 (2008).
4. Haramoto, E., Kitajima, M., Kishida, N., Konno, Y., Katayama, H., Asami, M., Akiba, M. Occurrence of pepper mild mottle virus in drinking water sources in Japan. *Appl Environ. Microbiol.* **79,** 7413-7418 (2013).
5. Green, H.C,. Dick, L.K., Gilpin, B., Samadpour, M., Field, K.G. Genetic markers for rapid PCR-based identification of gull, Canada goose, duck, and chicken fecal contamination in water. *Appl. Environ. Microbiol.* 78, 503-510 (2012).
6. Green, H.C., Haugland, R.A., Varma, M., Millen, H.T., Borchardt, M.A,. Field, K.G., Walters, W.A., Kinght, R., Sivaganesan, M., Kelty, C.A., & Shanks, O.C. Improved HF183 quantitative real-time PCR assay for characterization of human fecal pollution in ambient surface water samples. *Appl. Environ. Microbiol.* **80,** 3086-3094 (2014).
7. Stachler, E., Kelty, C., Sivaganesan, M,, Li, X., Bibby, K., & Shanks, O.C. Quantitative crAssphage PCR assays for human fecal pollution measurements. *Environ. Sci. Technol.* **51,** 9146-9154 (2017).
